# Integrated Microfluidic Chip Technology for Copper Ion Detection Using an All-Solid-State Ion-Selective Electrode

**DOI:** 10.3390/mi15010160

**Published:** 2024-01-21

**Authors:** Wenpin Zhang, Shuangquan Wang, Dugang Kang, Zhi Xiong, Yong Huang, Lin Ma, Yun Liu, Wei Zhao, Shouliang Chen, Yi Xu

**Affiliations:** 1 School of Optoelectronic Engineering, Chongqing University, Chongqing 400044, China; zhangwenpin@cqtj.org (W.Z.);; 2 Chongqing Special Equipment Inspection and Research Institute, Chongqing 401121, Chinaxiongzhi@cqtj.org (Z.X.); huangyong@cqtj.org (Y.H.); zhaowei@cqtj.org (W.Z.);; 3 Key Laboratory of Electromechanical Equipment Security in Western Complex Environment for State Market Regulation, Chongqing 401121, China

**Keywords:** all-solid-state ion-selective electrode, copper, microfluidic chip, detection system

## Abstract

This study involved the preparation of an all-solid-state ion-selective electrode (ASS-ISE) with copper and a poly(3,4-ethylenedioxythiophene) and polystyrene sulfonate (PEDOT/PSS) conversion layer through electrode deposition. The morphology of the PEDOT/PSS film was characterized, and the performance of the copper ion-selective film was optimized. Additionally, a microfluidic chip for the ASS-ISE with copper was designed and prepared. An integrated microfluidic chip test system with an ASS-ISE was developed using a self-constructed potential detection device. The accuracy of the system was validated through comparison testing with atomic absorption spectrophotometry (AAS). The experimental findings indicate that the relative standard deviation (RSD) of the integrated ASS-ISE with the copper microfluidic chip test system is 4.54%, as compared to the industry standard method. This value complies with the stipulated requirement of an RSD ≤ 5% in DL/T 955-2016.

## 1. Introduction

The quality of boiler water directly affects the safe use and economic operation of the boiler. Poor treatment and management of boiler water quality can easily lead to hazards such as boiler scaling, corrosion, and steam water co-evaporation [1]. The content of iron and copper metal ions in boiler water increases due to chemical and electrochemical corrosion. By detecting the content of metal ions in boiler water, it can indirectly be determined whether the boiler is corroded. This can prevent metal components from being damaged and iron scale from growing due to corrosion, thereby improving the service life of the boiler. Therefore, strengthening the monitoring of boiler water quality is an important link to ensuring the safety and economic operation of boilers, and it has very important practical significance.

At present, ultraviolet-visible (UV-Vis) spectra, graphite furnace atomic absorption spectrometry (GF-AAS), and chromatography can only detect copper ions in the laboratory, and there are shortcomings such as time-consuming sampling, complex preprocessing, and a long detection time [2,3,4]. However, when monitoring copper ions in boiler water, real-time on-site detection is most necessary. If a real-time and fast measurement mode can be adopted, it can save a lot of manpower, material resources, and financial resources and improve detection efficiency. Therefore, it is necessary to develop new heavy-metal-ion-monitoring instruments and propose new detection methods. The electrochemical sensing detection method has become one of the most promising on-site rapid detection methods [5].

Electrochemical sensors based on ion-selective electrodes (ISEs) have the advantages of high sensitivity, a fast response, and good selectivity, making for the on-site analysis of tracing metal ions in the environment. The all-solid-state ion-selective electrode is an ion-selective electrode, replacing the inner liquid with solid-state conductive electrode materials which are conducive to miniaturization, portability, and integration. The solid-state transfer layer not only eliminates the leakage at the solid–liquid interface but also helps overcome electrode miniaturization [6]. Therefore, all-solid-state ion-selective electrodes provide a new development opportunity for the research and development of ion-selective electrodes.

The all-solid-state selective electrode is composed of a conductive base layer, a solid-state conversion layer, and an ion-selective membrane layer [7]. At present, scholars at home and abroad are focusing their research on how to apply solid-state conversion layer materials to all-solid-state selective electrodes [8,9]. As the most classic conductive polymer until now, poly(3,4-ethylenedioxythiophene)/polystyrene sulfonate (PEDOT/PSS) shows suitable optical transparency, high conductivity, and good tensile properties compared to other polymer conductors. [10]. Zhao et al. [11] prepared an ASS-Cu^2+^-ISE by coating PEDOT with ion-electron conductivity on gold wire, which can efficiently convert ion signals into electrical signals. The test results showed that the test response range for copper ions is 2.5 × 10^−7^~2.5 × 10^−4^ mol·L^−1^ with a detection limit of 4.0 × 10^−8^ mol·L^−1^. Guzinski et al. [9] used PEDOT-C_14_ as a solid-state conversion material for hydrogen, sodium, and potassium ion-selective electrodes and prepared electrodes with a stable potential and short equilibrium time. The results showed that PEDOT-C_14_ had superhydrophobic properties which hindered the formation of selective membranes and solid-state conversion interlayer water layers. The Bobacka group [12] synthesized a 3D nano PEDOT/PSS film with a diameter of 750 nm using 3D nanosphere lithography and electrosynthesis techniques, and the prepared silver ion-selective electrode exhibited good repeatability. Zhao et al. [11] coated ion-selective membranes on gold microelectrodes modified with PEDOT/PSS and developed an all-solid-state ion-selective microelectrode (ISμE). Wan et al. [13] developed a microfluidic chip integrated with multiple ASS-ISEs based on PEDOT/PSS which can synchronously detect H^+^, NH^4+,^ and Ca^2+^ in water quality. The detection limits were 2.21 µmol·L^−1^, 8.4 µmol·L^−1^, and 3.44 µmol·L^−1^, respectively. Research has shown that PEDOT/PSS has advantages such as good electronic conductivity and dopable ion conductivity and can be used as a solid-state conductive layer for the study of many metal ions in solid-state electrodes. However, the practical application of PEDOT/PSS in microfluidic chip systems and on-site testing is relatively limited. This paper mainly studies the application of the redox-type conductive polymer poly(3,4-ethylenedioxythiophene)/polystyrene sulfonate (PEDOT/PSS) in all-solid-state copper ion-selective electrodes. The experimental results showed that the all-solid-state Cu^2+^-ISEs based on PEDOT/PSS have a fast response time and good selectivity. Further, an integrated all-solid-state ion-selective electrode microfluidic chip test system was built through a self-made potential detection device, and the accuracy of the built system can be used for the detection of copper ions in boiler water.

## 2. Materials and Methods

### 2.1. Laboratory Apparatus

The apparatus used in the study included an electrochemical workstation (CHI-660E) (Shanghai Chenhua Instrument Co., Ltd., Shanghai, China), electronic balance (FA2204B) (Shanghai Jingke Tianmei Scientific Instrument Co., Ltd., Shanghai, China), drying oven (GZX-9076MBE) (Shanghai Boxun Industrial Co., Ltd., Shanghai, China), scanning electron microscope (JSM-7800F) (JEOL, Tokyo, Japan), atomic absorption spectrophotometer (650P) (Jena, London, UK), and Smart-S ultrapure water machine (Shanghai Hetai Instrument Co., Ltd., Shanghai, China).

### 2.2. Experimental Reagents

EDOT (99%) and poly(4-sodium styrene sulfonate) (NaPSS) (Mw: ~70,000), polyvinyl chloride (PVC) with a K-value range of 72–71, o-nitrophenyloctyl ether (o-NPOE) with a purity of 98%, 2-mercaptobenzoxazole (2-MBA) with a purity of 98%, sodium tetrakis (3,5-bis(trifluoromethyl) phenyl)borate (NaTFPB) with a purity of 97%, tetrahydrofuran (AR), potassium chloride (AR), potassium ferricyanide (AR), copper sulfate pentahydrate (AR), zinc acetate (AR), nickel sulfate (AR), polydimethylsiloxane (PDMS), 1-dodecyl-3-methylimidazolium chloride, bis(2-ethylhexyl) sebacate (DOS), and ultrapure water were used as experimental reagents.

### 2.3. Preparation of Solutions

The EDOT/PSS membrane solution used in this study was a combination of 0.1 mol·L^−1^ NaPSS and 0.01 mol·L^−1^ EDOT.

The solution of the copper ion-selective membrane comprised 3% (*m*/*m*) of the copper ion carrier 2-MBA, 63.6% (*m*/*m*) of the plasticizer o-NPOE, 31.3% (*m*/*m*) of PVC, and 2.1% (*m*/*m*) of NaTFPB. The solvent used in the membrane was tetrahydrofuran.

The basic membrane solution of PVC contained 0.5–2% (*m*/*m*) IL, 32.5–33.5% (*m*/*m*) PVC, and 65.5–66.5% (*m*/*m*) DOS.

### 2.4. Preparation of the Integrated ASS-ISE Microfluidic Chip

Polyethylene terephthalate (PET) substrate material offers the benefits of high hardness, low bending resistance, and good flatness, enabling it to tightly adhere to PDMS cover sheets. In this study, PET was chosen as the electrode substrate for the integrated ASS-ISE microfluidic chip. The chip comprised a PET electrode substrate and a PDMS cover with a microreaction channel. An ASS selective electrode and an ASS reference electrode were integrated on a PET substrate. The specific preparation methods are as follows:

#### 2.4.1. Preparation of the ASS-ISE with Copper

The carbon paste conductive substrate for an ASS-ISE is fabricated through screen printing on a PET substrate. Subsequently, the carbon electrode is subjected to electrodeposition to modify the PEDOT/PSS conductive polymer film. Ultimately, the solid copper ion-selective electrode can be acquired through the modification of the copper ion-selective film via drip coating [14]. The ion-selective electrode utilizes silver pulp as the conductive electrode material.

#### 2.4.2. Preparation of the ASS Reference Electrode

A solid reference electrode with an Ag/AgCl conductive substrate is fabricated through screen printing on a PET substrate. The solid reference electrode can be produced through the modification of the PVC film with the ionic liquid 1-dodecyl-3-methylimidazolium chloride on the Ag/AgCl conductive substrate using the drip coating method [15]. The reference electrode employs silver pulp as the conductive medium.

#### 2.4.3. Preparation of the PDMS Cover

PDMS-coated surfaces containing micro-mixing and micro-detection channels are fabricated on a polymethyl methacrylate (PMMA) mold using the integral casting method [16].

### 2.5. Design and Preparation of the Ion-Selective Electrode Microfluidic Chip

**Figure 1:**
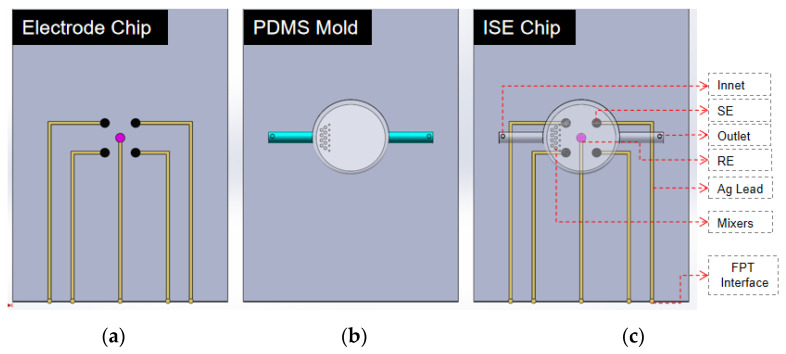
Detailed schematics of the microfluidic chip system for copper ion detection: (**a**) electrode microfluidic chip configuration; (**b**) PDMS mold design; and (**c**) ion-selective electrode microfluidic chip.

In response to the requirements for concurrent testing, the study presents a microfluidic chip configuration featuring simultaneous multi-electrode measurement, as depicted in Figure 1a. The electrode microfluidic chip comprises four carbon paste solid copper ion-selective electrodes, each with a 2 mm diameter, and an Ag/AgCl solid-state reference electrode, also with a 2 mm diameter. The distance between the selective electrodes is 6 mm, while the spacing between the reference electrode and the selective electrode is 8.5 mm.

**Figure 2:**
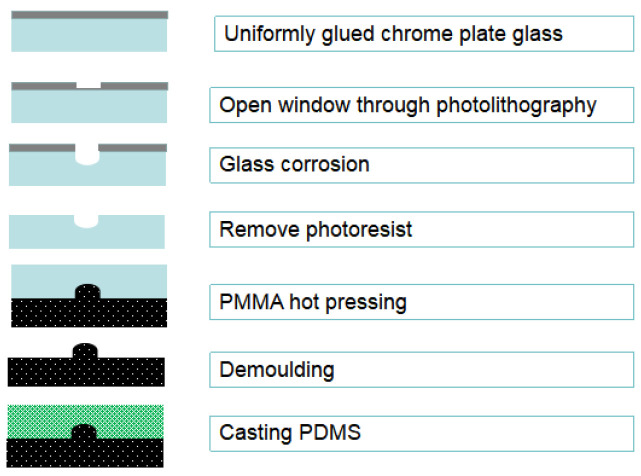
Schematic diagram of the PMMA mold and PDMS casting.

The PDMS mold, which is designed with PMMA as the substrate, is depicted in Figure 1b. The mold comprises a mixed microchannel sample, a sample detection tool, and a microchannel. The PMMA mold preparation process is shown in Figure 2.

The microfluidic chip PMMA mold is prepared by using the hot pressing method. After photolithography, the evenly glued chromium plate glass substrate is placed in a glass corrosion solution at 40 °C and corroded for 15 min. After photoresist removal and chromium removal, a glass substrate with microgrooves is obtained. The glass substrate with microgrooves is tightly adhered to the PMMA substrate, and the PMMA mold is pressed by a computerized laminating machine.

**Figure 3:**
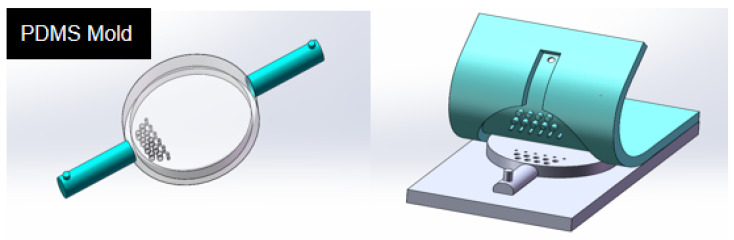
The preparation process of the PDMA cover.

The prepolymer and curing agent of PDMS are combined at a mass ratio of 10:1. Following the removal of bubbles through stirring, a PDMS cover with a microcavity and a micro-flow channel is cast at a temperature of 75 °C (Figure 3). The PDMS cover is prepared and integrated with the electrode microfluidic chip to create an integrated ion-selective electrode microfluidic chip (Figure 1c). The diameter of the PDMS microreactor is 8 mm, and the thickness of the microreactor is 1.5 mm. The spacing between the microfilters in the microreactor is about 1 mm.

### 2.6. Preparation and Morphological Characterization of the Conductive Polymer PEDOT/PSS

The preparation of PEDOT/PSS films on electrode substrates can be accomplished through electrochemical polymerization and direct drop coating methods. PEDOT/PSS can be prepared on carbon conductive substrates via electrochemical polymerization. In this study, the experiment involved electrochemical polymerization for 300 s and 714 s. The morphological characterization of the conductive polymer PEDOT/PSS was conducted using scanning electron microscopy.

### 2.7. Performance Optimization of the Copper Ion-Selective Membrane

The ion-selective membrane comprises an ion carrier, ion exchanger, plasticizer, and matrix material which serve the function of selectively allowing the passage of the target ion. Bakker et al. [17] discovered that the addition of a specific quantity of an ion exchange agent to the ion-selective film, which contains neutral ion carriers, enhances the selectivity of ion-selective electrodes. This enhancement in selectivity can lead to a reduction in the detection limit of the electrode. This study investigates the impact of the ion exchanger NaTFPB on the detection of electrode polarity.

### 2.8. Reproducibility Test of the ASS-ISE with Copper

The reproducibility of electrodes is a critical parameter for evaluating the practical application of electrodes. To assess the reproducibility of the electrode response, an ASS-ISE was repeatedly immersed in copper ion solutions with concentrations of 1 × 10^−4^ mol·L^−1^ and 1 × 10^−3^ mol·L^−1^. The repeatability of the electrode was assessed by recording the curve of potential changes.

### 2.9. Selectivity Test of the ASS-ISE with Copper

Selectivity plays a crucial role in determining the functionality of electrochemical sensors in real-world environments. Both the mixed solution method and the separated solution method are viable approaches for selectively testing ion-selective electrodes. This study employs the separate solution method to assess the selectivity of the solid-state copper ion-selective electrode that was prepared. The selection performance was assessed using the selectivity coefficient Equation (1).

(1) logKI,Jpot=(EJ−EI)ZiF2.303RT+logaIaJZiZj

In Equation (1), *E* represents the potential of the solution to be tested; *J* represents interfering ions; *I* represents the ion to be tested; *F* represents the Faradaic constant; *Z* represents the charge of the ion; *R* represents the gas constant; *T* represents the Kelvin temperature; and a represents ion activity in the solution.

### 2.10. Self-Made Potential Detection Device

This study addresses the limitation of laboratory electrochemical workstations, which are not suitable for on-site use, by proposing the design of a potential detection device capable of simultaneously measuring multiple potential differences on-site. Potential detection devices encompass signal processing circuits, signal acquisition modules, and data terminal modules.

## 3. Results and Discussion

### 3.1. Morphological Characterization of the Conductive Polymer PEDOT/PSS

**Figure 4:**
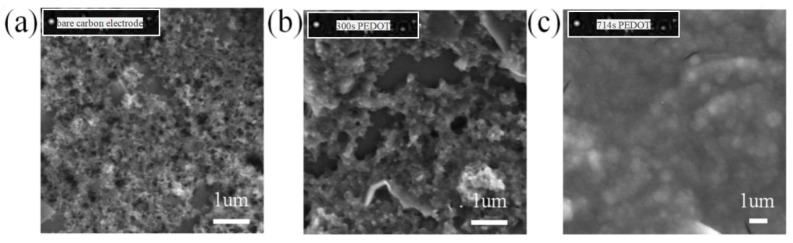
SEM images of PEDOT/PSS membrane development: (**a**) bare carbon electrode; (**b**) PEDOT/PSS deposited for 300 s; and (**c**) PEDOT/PSS deposited for 714 s.

Scanning electron microscopy (SEM) was used to analyze PEDOT/PSS conductive polymer films at various electrochemical deposition times, as depicted in Figure 4. The experimental findings indicate that the conductive polymer deposited for 714 s exhibits a uniform distribution and a larger surface area, which is advantageous for ion–electron conversion. Compared to bare carbon electrodes, the contact area between the conductive polymer PEDOT/PSS membrane and the ion-selective membrane is larger, thereby facilitating ion–electron conversion.

### 3.2. Optimization of the Copper Ion-Selective Membrane

**Figure 5:**
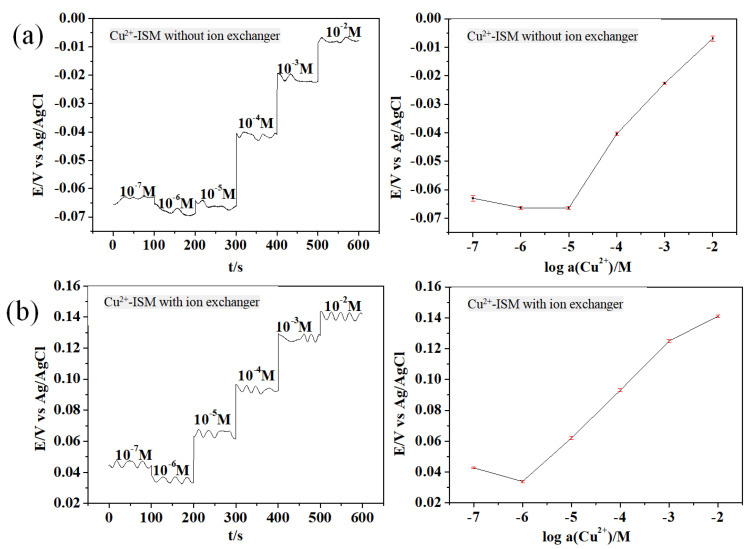
Comparative potential responses of the ASS-ISE with copper: (**a**) without an ion exchanger in the selective membrane and (**b**) with an ion exchanger in the selective membrane.

Based on the studies by Morteza [18] and Schwarz [19], ion-selective films for ASS copper ions were fabricated using 2-MBA as the ion carrier, o-NPOE as the plasticizer, and PVC as the membrane matrix. To investigate the impact of the ion exchanger NaTFPB on copper ion selectivity, the open-circuit potential results obtained from 1 × 10^−7^ mol·L^−1^ to 1 × 10^−2^ mol·L^−1^ copper sulfate solution are presented in Figure 5.

Figure 5a depicts the open-circuit potential response of solid copper ion-selective electrodes in the absence of the ion exchanger NaTFPB. The electrode potential stabilizes within 30 s and exhibits a linear response within the range of 1 × 10^−5^ mol·L^−1^ to 1 × 10^−2^ mol·L^−1^. The electrode exhibits a response slope of 20.7 mV/decade, and its detection limit is 1 × 10^−5^ mol·L^−1^. Figure 5b illustrates the open-circuit potential response of solid copper ion-selective electrodes containing the ion exchanger NaTFPB. The electrode potential stabilizes within 30 s and exhibits a linear response in the range of 1 × 10^−6^ mol·L^−1^ to 1 × 10^−2^ mol·L^−1^. The electrode response slope is 27.8 mV/decade, and the detection limit is 1 × 10^−6^ mol·L^−1^. The experimental results indicate that the detection limit performance of the solid copper ion-selective electrode containing the ion exchanger NaTFPB is superior. The detection limit is sufficient to meet the fundamental requirements for the on-site online detection of copper ions in boiler water.

### 3.3. Reproducibility of the ASS-ISE with Copper

**Figure 6:**
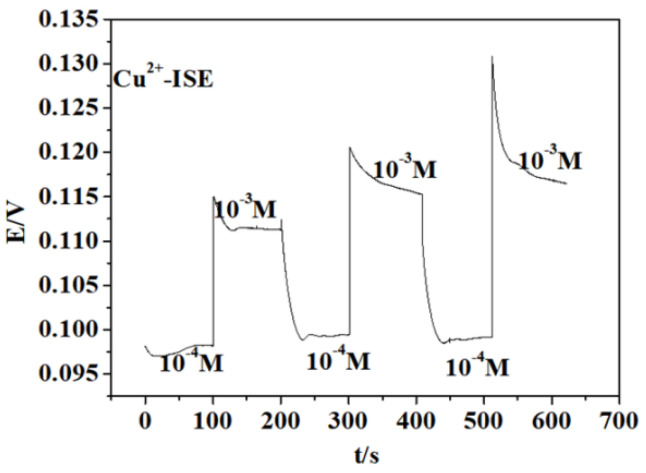
Reproducibility of the ASS-ISE with copper.

The reproducibility of electrodes is a critical parameter for assessing the suitability of the prepared electrodes for practical applications. If the reproducibility of the electrode is poor, this suggests that the electrode exhibits significant variation in response potential to ions of the same concentration over a brief timespan, rendering it impractical for calibration and unsuitable for accurate sample detection. To investigate the reproducibility of the electrode response, an ASS-ISE was subjected to repeated testing by immersing the electrode in copper ion solutions with concentrations of 1 × 10^−4^ mol·L^−1^ and 1 × 10^−3^ mol·L^−1^. The test results are presented in Figure 6. The experimental findings indicate that the potential of the all-solid copper ion electrode in a solution of the same concentration fluctuates within the range of 3 mV to 5 mV, demonstrating the electrode’s favorable repeatability.

### 3.4. Selectivity of the ASS-ISE

The selectivity of an ASS-ISE with copper was tested using the separation solution method. The electrode potential was measured sequentially in the presence of interference ions (e.g., Zn^2+^, Ni^2+^, Hg^2+^, Mg^2+^, Cd^2+^, and Co^2+^) and pure Cu^2+^ solutions with concentrations of 1 × 10^−4^ mol·L^−1^ and 1 × 10^−3^ mol·L^−1^. The selectivity coefficients of the ASS-ISE with copper are presented in Table 1. The test results suggest that when the concentration difference between interfering ions and copper ions is within two orders of magnitude, the interference between them is relatively minimal.

### 3.5. Construction of a Potential Detection Device

To rapidly and conveniently detect copper metal ions in boiler water, it is essential to not only incorporate ion-selective electrodes but also to develop appropriate detection circuits and optimize detection systems. While various robust electrochemical workstations are commonly utilized in laboratory settings for potential detection, they lack portability which makes them inconvenient for transportation.

Both the solid-state ion-selective electrode and the solid-state reference electrode produce direct current (DC) potential signals. Consequently, the development of a measurement circuit system capable of detecting potential signal disparities between two electrodes is imperative. This study presents the design of a detection circuit capable of concurrently measuring two potential differences. Its primary function is to filter out interference signals in the potential signal, distinguish the potential signals of the solid-state ion-selective electrode and the reference electrode, and amplify the signal. Simultaneously, to simplify the storage and processing of voltage signals produced by the detection circuit, the experiment also employs a data collector to gather voltage signals and transfer them to the computer for visualization and retention.

**Figure 7:**
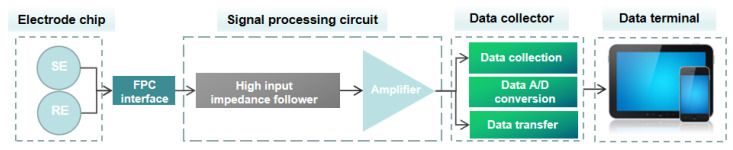
Schematic diagram of the proposed detection system.

The potential detection device, designed to measure multiple potential differences simultaneously, is depicted in Figure 7. It comprises a signal processing circuit, a signal acquisition module, and a data terminal module. The electrode chip is connected to the signal processing circuit device through the flexible printed circuit board (FPC). The signal processing circuit is capable of conducting potential difference signal testing between the ASS-ISE and the solid-state reference electrode. The signal acquisition module is capable of performing analog-to-digital (A/D) conversion and transmitting multiple DC potential differences. The data terminal module has the capability to store and process data. The signal processing circuit involves the acquisition of the potential difference signal between the solid-state ion-selective electrode and the solid-state reference electrode, while ensuring minimal interference with the testing system.

#### 3.5.1. Signal Processing Circuit

The ion-selective electrode testing system functions akin to a chemical primary battery, with the measured signal representing the voltage value of the primary battery. If the magnitude of the current passing through the electrode during measurement is substantial, it will induce polarization effects on the electrode, consequently impacting the electrode’s potential stability and rendering detection unfeasible. Hence, it is essential to guarantee minimal current flow through the ion-selective electrode during measurement. If the current passing through the ion-selective electrode is minimal, it necessitates an external measurement circuit with a high input impedance. A higher input impedance results in a reduced current passing through the ion-selective electrode, thereby minimizing its impact.

**Figure 8:**
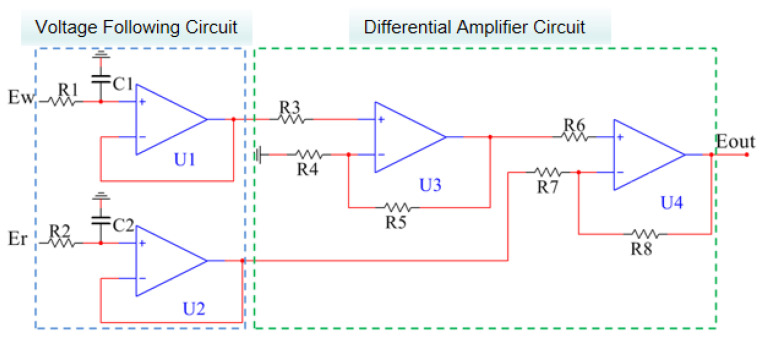
Circuitry blueprint of the signal processing system.

To mitigate the impact of the current on the potential of the ion-selective electrode, it is possible to connect the ion-selective electrode in series with the voltage-following circuit to enhance the impedance of the external detection circuit using a 1 × 10^12^ Ω high-input impedance ICL7621 operational amplifier. Simultaneously, to mitigate common-mode interference of signals between electrodes, the potential signal may be differentiated through the utilization of an ICL7621 operational amplifier to design a differential amplifier circuit (Figure 8).

#### 3.5.2. Data Collector

To achieve the acquisition of multiplex data, the data collector transmits multiple signals to the data terminal. The data collector comprises the 16-bit AD conversion chip AD7606, the main control chip STM32F205RBT6, and a USB interface. The collector is capable of simultaneously acquiring and transmitting 8 V signals. The data collector can efficiently transmit the collected voltage data to the host computer via USB 2.0. The primary purpose of the upper computer software is to assist users with transmitting instructions to the data collector via the USB 2.0 interface and managing the sampling interval and data collector’s timing. The upper computer software also serves the function of displaying and saving the data uploaded by the data collector, thereby providing users convenience in processing subsequent data.

#### 3.5.3. Data Terminal

The data terminal may take the form of a portable computer or an industrial tablet. The data terminal has the capability to regulate the frequency of acquisition and the duration of data collection, as well as to analyze and process the received data.

### 3.6. Construction and Testing of the Integrated ASS-ISE Microfluidic Chip Test System

#### 3.6.1. Construction of the Integrated ASS-ISE Chip Test System

**Figure 9:**
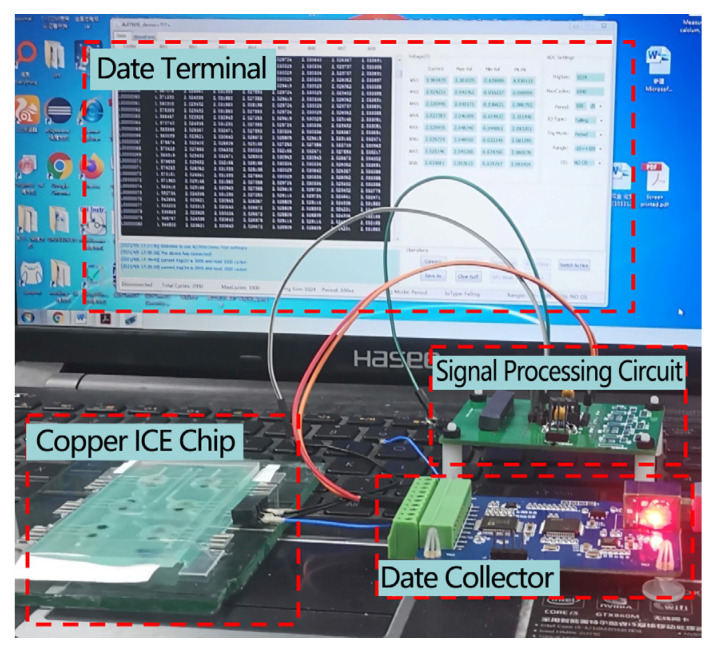
Configuration of the integrated ASS-ISE microfluidic chip test system.

The integrated microfluidic chip with the ion-selective electrode is linked to the FPC interface. The ASS-ISE and the ASS Ag/AgCl reference electrode are each connected to a self-assembled potential detection device. The potential difference is then collected using a data collector, and the resulting test data are analyzed and processed using a portable computer or an industrial flat plate. The integrated test system for the ASS-ISE microfluidic chip is illustrated in Figure 9. The integrated ASS-ISE chip test system is suitable for conducting on-site testing of boiler water quality.

#### 3.6.2. Stability Test of the Integrated ASS-ISE Microfluidic Chip Test System

To assess the viability of the self-constructed potential detection device, the potential response of a solid copper ion-selective electrode in a 1 × 10^−5^ mol·L^−1^ to 1 × 10^−3^ mol·L^−1^ copper sulfate solution was evaluated by comparing the self-made potential detection device with the Chenhua electrochemical workstation. The measurement results are presented in Table 2. The findings indicate that the self-constructed potential detection device exhibits a measurement value and error of less than 3 mV, while the electrochemical workstation demonstrates a similar level of accuracy. Additionally, the average error in the test results from the four electrodes is less than 2 mV. The test results indicate that the stability of the integrated all-state ion-selective electrode microfluidic chip test system meets the specified test requirements.

#### 3.6.3. Analysis of the Integrated ASS-ISE Microfluidic Chip Test System

The accuracy of the integrated ASS-ISE microfluidic chip test system was confirmed through comparative testing with the atomic absorption method. In the concentration range of 1 × 10^−7^ mol·L^−1^ to 1 × 10^−3^ mol·L^−1^, the average value of the four electrodes represents the test value of the integrated ASS-ISE microfluidic chip. The relationship is described by the linear equation Y = 20.3X + 202.6, with an R^2^ value of 0.997. The comparative test results of boiler water samples are presented in Table 3. Compared to the industry standard method, the relative standard deviation (RSD) of the integrated ASS-ISE microfluidic chip test system is 4.54%, thereby satisfying the RSD ≤ 5% requirement stipulated in DL/T 955-2016.

## 4. Conclusions

The ASS-ISE was fabricated using the electrodeposition method and incorporated a PEDOT/PSS conversion layer. The morphology of the PEDOT/PSS membrane was characterized, and the performance of the copper ion-selective film was optimized. Additionally, the reproducibility of the ASS-ISE was evaluated, and a microfluidic chip for the electrode was designed and prepared. The experimental findings indicate that the potential of the all-solid copper ion electrode fluctuates within the range of 3–5 mV in solutions of the same concentration, demonstrating good repeatability. An integrated microfluidic chip test system with an ASS-ISE was developed using a self-constructed potential detection device. The stability of the self-constructed potential detection device was confirmed through comparison with the electrochemical workstation. The experimental findings indicate that the stability of the integrated ASS-ISE microfluidic chip test system satisfies the test requirements. Ultimately, the system’s accuracy was confirmed through a comparison test using atomic absorption spectrophotometry. The experimental findings indicate that the integrated ASS-ISE microfluidic chip test system demonstrates an RSD of 4.54% in the concentration range of 1 × 10^−7^ mol·L^−1^ to 1 × 10^−3^ mol·L^−1^ when compared to the industry standard method. This result complies with the RSD ≤ 5% requirement specified in DL/T 955-2016. Therefore, the integrated ASS-ISE microfluidic chip test system is suitable for the on-site detection of boiler water quality.

## Figures and Tables

**Table 1:** The selectivity coefficient of the ASS-ISE with copper.

Interfering Ions	Cu^2+^ Ion-Selective Electrode
Cu^2+^	/
Zn^2+^	−3.11
Ni^2+^	−2.34
Hg^2+^	−2.81
Mg^2+^	−3.73
Cd^2+^	−2.04
Co^2+^	−2.44

**Table 2:** Comparative analysis between the self-made potential detection device and the Chenhua electrochemical workstation.

Cu^2+^ Concentration	Self-Made Potential Detection Device Test Voltage (mV)				Chenhua Workstation Test Voltage (mV)			
	ISE1	ISE2	ISE3	ISE4	ISE1	ISE2	ISE3	ISE4
1 × 10^−5^ mol·L^−1^	112.6	113.9	112.8	111.8	114.4	115.1	114.9	113.8
1 × 10^−4^ mol·L^−1^	130.9	131.7	129.6	132.5	131.9	133.0	131.3	133.3
1 × 10^−3^ mol·L^−1^	150.3	151.5	149.6	151.9	152.0	153.3	152.5	152.9

Note: The SE electrode located in the upper left corner of the electrode microfluidic chip is ISE1, which rotates clockwise to ISE2, ISE3, and ISE4.

**Table 3:** Comparative analysis of copper ion concentration measurements in boiler water.

Samples	AAS-ISE		AAS
Boiler water Cu^2+^ test value	1.53 × 10^−6^ mol·L^−1^	97.16 μg·L^−1^	92.84 μg·L^−1^
	1.47 × 10^−6^ mol·L^−1^	93.34 μg·L^−1^	87.81 μg·L^−1^
	1.45 × 10^−6^ mol·L^−1^	92.08 μg·L^−1^	89.67 μg·L^−1^
Boiler water Cu^2+^ average		94.19 μg·L^−1^	90.11 μg·L^−1^
RSD	4.54%		

## Data Availability

The datasets utilized and/or examined in this study can be obtained from the corresponding author upon making a reasonable request.
